# The *hOGG1* Ser326Cys Polymorphism Is Not Associated with Colorectal Cancer Risk

**DOI:** 10.2188/jea.17.156

**Published:** 2007-09-01

**Authors:** Hye-Won Park, Il-Jin Kim, Hio Chung Kang, Sang-Geun Jang, Sun-A Ahn, Jin Soo Lee, Hai-Rim Shin, Jae-Gahb Park

**Affiliations:** 1 Korean Hereditary Tumor Registry, Cancer Research Institute and Cancer Research Center, Seoul National University.; 2 Research Institute and Hospital, National Cancer Center.

**Keywords:** Clorectal Neoplasms, Polymorphism, Single Nucleotide, *hOGG1*, DNA Repair

## Abstract

**BACKGROUND:**

Little is known about the genetic risk factors associated with colorectal cancer. Although the Ser326Cys polymorphism of 8-oxoguanine DNA glycosylase (*hOGG1*) is consistently associated with a range of cancers, there is no consensus regarding this polymorphism and colorectal cancer risk.

**METHODS:**

In the present study, conducted in a Korean population, we used the TaqMan assay to investigate whether the *hOGG1* Ser326Cys polymorphism was associated with colorectal cancer in 439 colorectal cancer patients and 676 healthy normal controls. We also examined whether the *hOGG1* Ser326Cys polymorphism is associated with tumor location, microsatellite instability (MSI) status and tumor-node-metastasis (TNM) stage in colorectal cancer.

**RESULTS:**

We found no significant difference between the cancer and control populations in terms of genotype distribution (CC, CG and GG). In addition, we found no association between the *hOGG1* Ser326Cys polymorphism and cancer risk, MSI status, TNM stage or tumor location in colorectal cancer patients.

**CONCLUSIONS:**

These results suggest that unlike for other cancer types, the hOGG1 Ser326Cys polymorphism is not a major genetic risk factor for colorectal cancer.

Although colorectal cancer is a highly malignant disease, little is known about the genetic factors that could alter the physiology of the colorectum and increase the risk of colorectal cancer development. Inherited deficiencies in the DNA repair system are associated with increased cancer susceptibility,^1^ and DNA repair gene polymorphisms may increase the risk of colorectal cancer. Oxidative DNA damage induced by reactive oxygen species (ROS) can cause mutations that are substrates for DNA repair systems in prokaryotes and eukaryotes.^2^ Base excision repair (BER) pathway genes are involved primarily in repair of ROS-associated DNA damage.^3^ The 8-oxoguanine DNA glycosylase (*OGG1*) gene, a BER gene family member, encodes a DNA repair enzyme that can directly remove 8-hydroxyguanine (8-oxoG), a major base lesion produced by ROS causing G:C to T:A transversions.^4^ The *hOGG1* gene is located on human chromosome 3p25, a region frequently missing in various cancers, particularly lung and kidney tumors, which show loss of heterozygosity of markers.^5^

The 1245C>G polymorphism (Ser326Cys) is a well-known *hOGG1* gene polymorphism that results in an amino acid substitution from serine to cystein in codon 326. Compared to the *OGG1*-Ser326 protein, the *OGG1*-Cys326 protein was shown to be less able to suppress spontaneous mutations in an Escherichia coli strain defective in 8-oxoG repair.^6^ Recently, the *hOGG1* Ser326Cys variant has been consistently associated with an increased risk for a range of cancers.^7^ The *hOGG1* Ser326Cys polymorphism is associated with an increased risk of lung, esophageal and prostate cancer, whereas no association has been observed with breast cancer or basal cell carcinoma.^8^^-^^13^ The issue of whether the *hOGG1* Ser326Cys polymorphism is associated with colorectal cancer remains controversial.^14^^,^^15^

In the present study, we investigated whether the *hOGG1* Ser326Cys polymorphism was associated with colorectal cancer risk by genotyping over 1,000 colorectal cancer patients and normal controls from the Korean population. In addition, we investigated whether the Ser326Cys polymorphism and colorectal cancer risk might be modulated by clinico-pathological characteristics such as tumor location, tumor-node-metastasis (TNM) stage or microsatellite instability (MSI) status.

## METHODS

### Samples and DNA Extraction

We collected 373 colorectal cancer patients from Seoul National University Hospital and the remaining 66 cases from National Cancer Center. All 676 healthy controls were collected from National Cancer Center (Korea). Eight out of 439 colorectal cancer patients were HNPCC cases and the others are all sporadic colorectal cancers. All colorectal cancer patients were pathologically diagnosed as colorectal cancers and surgically operated. Normal controls were selected from cancer-free samples enrolled from the Cancer Cohort Study Branch of the National Cancer Center. The mean ages of the cases and controls were 59.3 ± 12.5 and 50.3 ± 11.7 years, respectively. The case group comprised 283 males and 156 females, while the control group consisted of 400 males and 276 females. Of the 439 colorectal cancer subjects, 423 were able to be classified according to TNM. DNA was extracted from normal colorectal tissue of cancer patients, and from peripheral blood lymphocytes of normal controls. Total genomic DNA was extracted using Trizol^®^ reagent (Invitrogen, CA, USA) according to the manufacturer's instructions.

### Genotyping

The *hOGG1* Ser326Cys polymorphism (rs1052134) was genotyped using the TaqMan^®^ assay (7900HT, Applied Biosystems., CA, USA).^16^ Briefly, 5 μL reactions contained 2X Universal PCR Master Mix, 900 μM primers, 200 μM probe and 10 ng genomic DNA. Cycling conditions were as follows; 50°C for 2 min, 95°C for 10 min, 40 cycles of 95°C for 15 sec, and 60°C for 1 min performed in 384-well plates. For quality control purposes, each assay run included all three genotype controls plus non-template controls. The *hOGG1* Ser326Cys genotyping was analyzed using allele discrimination plots using the Sequence Detection Software (SDS) program (version 5.0, Applied Biosystems, Foster City, CA). A part of samples were randomly confirmed using direct sequencing (ABI 3100 DNA sequencer, Perkin-Elmer, CA, USA).

### Microsatellite Instability Analysis

Of the 439 cancer patients, 273 had colon cancers in the distal region, while the remaining 166 had proximal region tumors. MSI analysis was not possible for 12 cancer patients due to poor quality samples, and TNM stage was assessed in 423 patients (Table 2). MSI analysis was performed using DHPLC (Denaturing High Performance Liquid Chromatography) and two microsatellite markers (BAT-25, BAT-26).^17^ Briefly, PCR reactions were performed as follows: 5 min at 94°C; 30 sec at 55°C (BAT-25) or 50°C (BAT-26); and 1 min at 70°C for 35 cycles, followed by a final elongation of 7 min at 70°C. The 5 μL PCR product was run under non-denaturing conditions (50°C) in DHPLC.

**Table 2.** Association between Ser326Cys genotypes and clinicopathological colorectal cancer features.

	n=439	Ser/Ser (%)	Ser/Cys (%)	Cys/Cys (%)	P for trend
Tumor location					
Distal	273	57 (20.9)	144 (52.7)	72 (26.4)	
Proximal	166	34 (20.5)	76 (45.8)	56 (33.7)	0.232
MSI status					
MSS*	369	77 (20.9)	190 (51.5)	102 (27.6)	
MSI*	58	13 (22.4)	26 (44.8)	19 (32.8)	0.619
TNM stages					
I	17	2 (11.8)	10 (58.8)	5 (29.4)	
II	163	36 (22.1)	89 (54.6)	38 (23.3)	
III	151	34 (22.5)	75 (49.7)	42 (27.8)	
IV	92	15 (16.3)	43 (46.7)	34 (37.0)	0.381

* MSS, microsatellite stability; MSI, microsatellite instability

### Statistical Analysis

Chi-square or Fisher's exact tests were used to assess differences in genotype distribution. Genotype-specific risks were estimated as odds ratios (OR) with 95% confidence intervals (CI) using logistic regression models. All statistical tests were performed using SPSS^®^ software (version 12.0, SPSS Inc, Chicago, IL). A p value of less than 0.05 was considered to indicate a significant difference.

## RESULTS

We found that for colorectal cancer patients, 91/439 (20.7%) were homozygous for the C allele, 220/439 (50.1%) were heterozygous (C/G), and 128/439 (29.2%) were homozygous for the G allele. For normal controls, 120/676 (17.7%) were homozygous for the C allele, 333/676 (49.3%) were heterozygous (C/G), and 223/676 (33.0%) were homozygous for the G allele. The normal control genotype distributions were in Hardy-Weinberg equilibrium. Consistent with a previous report, we found that the G allele for the Ser326Cys polymorphism was more prevalent in Asian populations than in Caucasian populations.^18^ Analysis showed there was no association between the Ser326Cys polymorphism and colorectal cancer risk (Table 1). Analysis of Cys/Cys carriers versus combined Ser/Ser and Ser/Cys carriers also showed there was no association between Cys/Cys carriers and colorectal cancer risk (*p*=0.289) (Table 1). We stratified cases into HNPCC and non-HNPCC and re-analyzed to see any relationship between *hOGG1* Ser326Cys polymorphism and stratified cases. There was no significant relationship between Ser326Cys and stratified cases (data not shown).

**Table 1.** *hOGG1* Ser326Cys polymorphisms in colorectal cancer patients and control subjects in this study and previous reports.

	Number (%)		odds ratio (95% CI)	*P*	Kim et al.*	Hansen et al.*
	
	controls (n=676)	cases (n=439)			controls/cases (%)	
Ser/Ser	120 (17.7)	91 (20.7)	1.00 (reference)	0.454	21.1/19.2	52.5/61.2
Ser/Cys	333 (49.3)	220 (50.1)	0.89 (0.64-1.25)	0.502	53.0/52.8	41.4/33.3
Cys/Cys	223 (33.0)	128 (29.2)	0.79 (0.55-1.14)	0.215	25.9/28	6.1/5.5
Combined genotype						
Ser/Ser + Ser/Cys	453 (67.0)	311 (70.8)	1.00 (reference)			
Cys/Cys	223 (33.0)	128 (29.2)	0.86 (0.66-1.13)	0.289		

Odds ratios adjusted by logistic regression for age and sex.

* : genotype distribution of colorectal adenocarcinomas

Kim et al. from reference 14; Hansen et al. from reference 15.

CI: confidence interval

We investigated whether there was any association between the Ser326Cys polymorphism and clinico-pathological features such as tumor location, TNM stage and MSI status. This analysis found no association between the polymorphism and MSI status (p=0.619) (Table 2), tumor location (p=0.232) or TNM stage (p=0.381) (Table 2).

## DISCUSSION

It has been suggested that the *OGG1*-Cys326 protein confers cancer susceptibility because it is less able than *OGG1*-Ser326 to prevent 8-oxoG-associated mutagenesis.^19^^,^^20^ However, an *in vitro* study of endogenous 8-oxoG-specific lyase activity in human colorectal carcinoma tissues found no significant difference in lyase activity between carriers of different *hOGG1* genotypes.^21^ The apparent controversy surrounding the functional consequence of the *hOGG1* Ser326Cys polymorphism might be associated with the studies indicating that the polymorphism is associated with increased risk of lung, esophageal and prostate cancer,^9^^-^^11^ but not breast cancer or basal cell carcinoma.^12^^-^^13^

The present study investigated *hOGG1* polymorphism in colorectal cancers, and found no association between the Ser326Cys polymorphism and colorectal cancer risk. Of the two similar previous studies, Kim et al^14^ also found no association between the Ser326Cys polymorphism and colorectal cancer risk. In contrast to these findings, a study of over 1,000 Norwegians by Hansen et al^15^ concluded the *hOGG1* Ser326Cys variant was indeed associated with colon cancer. They found that carriers of the rare Cys allele had a lower risk of colorectal cancer, particularly of adenocarcinoma (166 patients), but not a lower risk of adenoma. They concluded that the *hOGG1* S326C polymorphism may be protective against colorectal cancer development but not carcinogenesis.^15^ These findings are not consistent with those of the present study involving over 400 carcinoma cases in the Korean population.

Previous reports suggest that the frequency of the *hOGG1* Ser326Cys polymorphism depends on race and ethnicity.^7^^,^^8^ Studies involving a number of cancer types have shown that the Asian population tends to have a greater proportion of GG(Cys/Cys) genotypes than the Western population.^7^^,^^11^^,^^18^ Consistent with those reports, the present study found a higher proportion of Cys/Cys genotype frequencies in this Korean population (33.0% in controls, 29.2% in cases) compared to that reported for Western populations, in particular Norwegians (12.1% in controls, 7.9% in cases).^15^ Thus, it appears the ^hOGG1^ S326C polymorphism may be differently distributed among different ethnic groups. These findings indicate the need for studies examining whether the Ser326Cys polymorphism is associated with colorectal cancer in larger Asian populations such as the Chinese or Japanese. Moreover, *hOGG1* Ser326Cys polymorphism may not be a risk factor of colorectal cancers in the Korean population.

No study on the relationship between MSI and *hOGG1* Ser326Cys polymorphism was reported in human cancers. Interestingly, it was reported that monoallelic MYH, one of BER genes, germline mutations showed a negative correlation with MSI in colorectal cancer patients.^22^ It was also reported that BER deficiency was rarely accompanied by CIN (Chromosomal instability) or MSI.^23^ Another study showed that *MLH1* −93G>A polymorphism was associated with an increased risk of MSI-H colorectal cancer.^24^ Thus, we investigated whether MSI might be associated with *hOGG1* Ser326Cys polymorphism in colorectal cancers and found no significant result. This result suggests that there is no impact of *hOGG1* Ser326Cys polymorphism to the MSI.

It is important to consider environmental factors in a case-control genotyping analysis. However, we could not get information on the alcohol consumption, physical exercise, smoking status, and folate consumption in our samples. This is a limitation on this study.

In summary, the present large-scale study of Koreans found that the Ser326Cys polymorphism was not associated with colorectal cancer development. Thus, although the *hOGG1* Ser326Cys polymorphism is reported to be a risk factor in some cancer types, it does not appear to play a major role in colorectal cancer development, at least in the Korean population.
